# The relationship among psychosocial adaptation, medication adherence and quality of life in breast cancer women with adjuvant endocrine therapy

**DOI:** 10.1186/s12905-022-01722-0

**Published:** 2022-04-27

**Authors:** Haoran Jiang, Yu Dong, Wei Zong, Xiu-jie Zhang, Hui Xu, Feng Jin

**Affiliations:** 1 Department of Breast Oncology, The First Affiliated Hospital of China Medical University, Shenyang, China; 2 Nursing Department, The First Affiliated Hospital of Dalian Medical University, Dalian, China; 3 Department of Oncology, Cancer Hospital of China Medical University, Liaoning Cancer Hospital & Institute, Shenyang, China; 4 Department of Breast Oncology, The First Affiliated Hospital of China Medical University, No. 155, North Street, Heping District,, Shenyang, 110001 Liaoning Province China

**Keywords:** Breast cancer women, Adjuvant endocrine therapy, Psychosocial adaptation, Medication adherence, Quality of life

## Abstract

**Background:**

Patients undergoing endocrine therapy for breast cancer often suffer from poor psychosocial adaptation, low compliance with endocrine therapy and poor quality of life. However, the relationship among the three is not completely clear. The aims of this study were to investigate the status of psychosocial adaptation (PSA), medication adherence and quality of life (QOL) in breast cancer women with adjuvant endocrine therapy (AET), and to analyze the influencing factors of QOL and explore the relationship among them.

**Methods:**

346 breast cancer women were selected who underwent endocrine therapy after surgery, and data collected by the general information questionnaire, the PSA questionnaire among breast cancer women with AET, Morisky Medication Adherence Scale and The Functional Assessment of Cancer Therapy-Breast (FACT-B). The relationship among the variables was investigated by univariate analysis, multiple stepwise regression analysis and mediating effect analysis.

**Results:**

The scores of PSA, medication adherence and QOL were slightly above the medium level. Univariate analysis showed that there were significant differences in QOL among breast cancer women of AET with different types of exercise, medical payment methods, discomfort symptoms (headache, hypomnesis, arthralgia, perturbation), type of discomfort symptoms, medication adherence and PSA; Multi-factor analysis showed that PSA and medication adherence were the influential factors of QOL; mediating effect showed that medication adherence played a partial mediating role in PSA and QOL.

**Conclusion:**

The QOL of breast cancer women with AET will be directly affected by PSA. Medication compliance has a weak mediating effect in the indirect impact of PSA on the QOL. In the future, clinical nursing work should take targeted measures to improve the PSA level of patients, and effectively improve the compliance of patients with medication, so as to better improve the QOL of breast cancer women.

## Introduction

Breast cancer is the most common malignancy in women worldwide [1], and also causes the largest number of deaths among women worldwide [2]. According to the latest global cancer burden data released by the World Health Organization/International Agency for Research on Cancer (WHO/IARC) in December 2020[3], the number of new cases of breast cancer reached 2.26 million (accounting for 11.7% of the total number of newly diagnosed cancers), becoming the largest cancer in the world. Breast cancer is also the fifth leading cause of cancer deaths worldwide, accounting for more than 680,000 deaths (6.9%). In China, the number of new cases of breast cancer reached 426,000, with 117,000 deaths. The incidence and mortality rate of breast cancer are still rising [1, 3].

The vast majority of breast cancer women (BCW) do not have metastasis at diagnosis (90%) [4] and the goal of treatment is primarily to eliminate the tumor and prevent recurrence. More than 2/3 of breast cancers are estrogen receptor (ER) positive and/or progesterone receptor (PR),and hormone-dependent breast cancer women are sensitive to endocrine therapy drugs [5]. In breast cancer women who have completed the basic treatment of chemotherapy, radiotherapy or surgery, endocrine therapy reduces the recurrence rate of breast cancer by 34–45% and the mortality rate by 29–34% [6, 7].

Although endocrine therapy can reduce the recurrence rate and death rate, in clinical practice, BCW often do not completely adhere to the prescribed medication for some reason, or even stop treatment at an early stage. The rate of non-adherence with endocrine therapy is between 13.4 and 73% [8–10]. Studies have shown that 32% of endocrine therapy patients stopped endocrine therapy at the early stage of treatment (24 months) [11]; Patients treated with endocrine therapy showed significant discontinuities at year 1–2 and again at year 3 [9].

In addition, BCW during long-term endocrine therapy, there will be anxiety, fear, worry about metastasis or recurrence, social communication ability, sexual dysfunction and other psychological and social changes. In the face of these changes, breast cancer women often suffer from psychosocial maladjustment, which seriously affects the treatment, prognosis, compliance with endocrine therapy and the quality of life of patients. Therefore, paying attention to the psychosocial adaptation (PSA) of BCW undergoing endocrine therapy and taking corresponding nursing intervention measures can help patients improve their adherence with endocrine therapy, so as to prolong survival time and improve the quality of life (QOL). This study investigated the status of PSA, medication adherence and QOL of BCW with AET, and explored the relationship between PSA and medication adherence and QOL, so as to provide a basis for improving medication adherence and QOL of patients with AET.

## Methods

### Design

This study was a cross-sectional design.

### Study population

Subjects were randomly selected from breast cancer women who met the following inclusion and exclusion criteria and received endocrine therapy after surgery in the breast surgery ward of the First Affiliated Hospital of China Medical University and Liaoning Provincial Cancer Hospital between 2010 and December 2020.

Inclusion criteria: positive for the ER and/or PR; the pathological stage was I ~ IIIA; complete radiotherapy and chemotherapy, take estrogen receptor modulators (such as tamoxifen, etc.) or third-generation aromatase inhibitors (such as letrozole, anastrozole, exemestane, etc.); breast cancer women above 18 years old; able to communicate normally; volunteer to participate in this survey.

Exclusion criteria: patients with history of other malignant tumors; during the treatment of breast cancer recurrence or recurrence.

Sample size: 10 times the independent variable is considered the most appropriate. In this study, there were 28 independent variables. considering the cases of invalid responses, 20% is expanded on this basis, at least 336 questionnaires need to be issued.

## Data collection

### Questionnaire

#### General information questionnaire

Demographics data include age, height, weight, occupational status, education level, marital status, medical payment method, etc. Information related to disease includes time of diagnosis, time of operation, endocrine therapy medicine, and discomfort symptoms during taking.

### The psychosocial adaptation questionnaire among BCW with AET

It was used to assess the psychosocial adaptation of endocrine therapy of breast cancer women, including four dimensions, a total of 16 items, namely the emotional dimension (6 items), self-cognitive (5 items), self-image (3 items) and social dimension (2 items) [12]. The answer options were rated by a 5-point Likert-type scale: all the time, often, sometimes, rarely, and never. Forward items are scored on a scale of 0–4, and 5 items of self-cognition are scored in reverse. The lowest score was 0 and the highest was 64. The higher the score, the better the psychosocial adaptation. The Cronbach’s alpha coefficient of the questionnaire was 0.876.

### Morisky Medication Adherence Scale

It was developed by Morisky [13] in 1986 and first used to measure the medication adherence of patients with hypertension. Since no specific diseases were involved, it was widely used to measure the medication adherence of patients taking long-term medication. In 2008, Morisky [14] equal formed a widely used 8-item medication adherence questionnaire based on the original scale. The Cronbach's alpha coefficient, sensitivity and specificity of the questionnaire were 0.83, 0.93, and 0.53. Zhang Siyu etc. conducted a Chinese version of the questionnaire, and the Cronbach's alpha coefficient of the Chinese version of the questionnaire was 0.64. The total score of the scale is 8 points, 8 is high medication adherence, 6–8 is medium medication adherence, and 0–6 is low medication adherence. Adherence rate = number of respondents with more than 6 points/total number of respondents × 100%.

### The Functional Assessment of Cancer Therapy-Breast (FACT-B)

36 items will be divided into five areas [15], namely: physical well-being (7 items), social/family well-being (7items), emotional well-being (6 items), functional well-being (7 items) and additional concerns (9 items), all items are set in a hierarchical way, which can be divided into five grades: not at all (0), a little (1), some (2), quite (3), and very (4). In the scoring process, the forward items are directly scored from 0 to 4 points, and the reverse items (the larger the number of answer options, the worse the quality of life) are scored in reverse, with four points for those who fill in the first level, three points for those who fill in the second level, and so on. Among them, GP1 ~ GP7, GE1, GE3 ~ GE6, B1 ~ B3, B5 ~ B8 are reverse items, and the rest are forward items. According to this standard, each item, each field and total table score can be calculated respectively. The Cronbach's alpha coefficient for the whole questionnaire is 0.9, and for each dimension ranged from 0.61 to 0.84. In this study, the total score was defined as patients’ quality of life.

### Investigation methods

The contents of the general information shall be obtained from the registration book of the department. Questionnaire star and paper questionnaire are adopted to ensure the quality of questionnaire filling by setting mandatory items and filling time. The telephone follow-up of paper questionnaire was conducted by the uniformly trained research team. Before the investigation, the purpose, significance, and requirements of the study were explained to the patients, so as to obtain their cooperation and promise to keep their data absolutely confidential.

### Statistical analysis

Each questionnaire was reviewed and entered Excel 2016 by two researchers, and SPSS26.0 software was used for data processing and statistical analysis. The quantitative data of normal distribution were described by mean ± standard deviation (x ± s). The qualitative data were described by frequency and percentage. In the univariate analysis, the independent sample T test was used when the classified variables were normally distributed, and the Mann–Whitney U test or Kruskal–Wallis H test was used for non-normal distribution. In the multivariate analysis, the meaningful variables in the univariate analysis were used as independent variables, and the scores of the QOL measurement scale for breast cancer women were used as the dependent variables, and multiple linear regression analysis was performed. SPSS process plug-in and Amos are used as the mediating effect model.

### Ethics

This study was approved by the ethics committee at First Affiliated Hospital of China Medical University (No. 2016–5-2). Participants have adequate information about the purpose, methods, and processes of the study. Participants had the right to choose freely and could choose to participate or withdraw from the study. All participants signed the informed consent.

## Results

In this study, 354 questionnaires were sent out and 346 effectively received with effective recovery rate of 97.74%. The age range of the patients ranged from 29 to 82 years, with an average age of 50.46 ± 9.23 years. Among them, 45–54 years old accounted for the largest proportion (40.75%), normal weight (45.38%), the vast majority were married (84.97%), junior high school and below (27.75%), retired (41.04%), irregular exercise (50.29%), urban basic medical insurance (77.46%), other characteristics are shown in Table 1.

**Table 1:** Data characteristics of research samples and results of univariate analysis (n = 346)

	N (%)	Quality of life	
		Mean ± SD	p
*Age (years)*			0.138
< 45	88 (25.43)	99.65 ± 19.99	
45–54	141 (40.75)	104.41 ± 19.00	
≥ 55	117 (32.82)	103.46 ± 18.17	
*BMI*			0.082
Low weight	6 (1.73)	116.67 ± 22.23	
Normal weight	157 (45.38)	101.22 ± 19.34	
Overweight	154 (44.51)	104.28 ± 18.67	
Obesity	29 (8.38)	101.55 ± 17.77	
*Marital status*			0.421
Married	294 (84.97)	103.26 ± 19.23	
Unmarried	9 (2.60)	93.11 ± 20.31	
Divorced	27 (7.80)	101.67 ± 18.43	
widowed	16 (4.62)	103.44 ± 15.27	
*Education level*			0.144
Junior high school and below	96 (27.75)	100.73 ± 20.16	
High school/technical secondary school	94 (27.17)	100.59 ± 20.49	
Junior college	71 (20.52)	108.28 ± 16.42	
Bachelor's degree	78 (22.54)	103.17 ± 17.76	
Master degree or above	7 (2.02)	105.14 ± 13.21	
*Professional*			0.242
Retired	142 (41.04)	104.19 ± 17.53	
Did not work	92 (26.59)	100.25 ± 20.88	
Temporary worked	13 (3.76)	96.46 ± 19.71	
Work as usual	99 (28.61)	104.28 ± 19.06	
*Exercise*			0.000**
Regular exercise	150 (43.35)	107.47 ± 17.76	
Irregular exercise	174 (50.29)	100.76 ± 18.76	
No exercise	22 (6.36)	88.32 ± 20.00	
*Medical payment method*			0.013*
Self-pay	16 (4.62)	94.00 ± 17.09	
Urban basic medical insurance	268 (77.46)	104.28 ± 18.87	
Commercial medical insurance	5 (1.45)	83.80 ± 14.02	
Cooperative medical insurance	50 (14.45)	99.90 ± 20.09	
Others	7 (2.02)	104.43 ± 11.79	
*Endocrine drug*			0.853
Tamoxifen	128 (36.99)	103.97 ± 17.35	
furlong (Letrozole)	76 (21.97)	100.87 ± 19.65	
Exemestane (Arnoxine)	45 (13.01)	102.91 ± 18.10	
Torremifene (fareston)	45 (13.01)	105.29 ± 18.06	
Others	52 (15.02)	101.02 ± 23.45	
*Discomfort symptoms*			
Nausea			0.851
Yes	14 (4.05)	101.29 ± 20.55	
No	332 (95.95)	102.95 ± 18.99	
*Headache*			0.035*
Yes	28 (8.09)	96.43 ± 19.34	
No	318 (91.91)	103.45 ± 18.92	
*Hot flash*			0.341
Yes	164 (47.40)	101.70 ± 19.10	
No	182 (52.60)	103.94 ± 18.95	
*Memory loss*			0.013*
Yes	162 (46.82)	99.88 ± 20.05	
No	184 (53.18)	105.52 ± 17.72	
*Arthralgia*			0.003**
Yes	138 (39.88)	98.96 ± 19.58	
No	208 (60.12)	105.48 ± 8.24	
*Ankylosis*			0.108
Yes	124 (35.84)	100.31 ± 20.26	
No	222 (64.16)	104.32 ± 18.19	
*Perturbation*			0.032*
Yes	46 (13.29)	97.57 ± 18.02	
No	300 (86.71)	103.69 ± 19.07	
*Hyperplasiaendometrii*			0.159
Yes	40 (11.56)	99.55 ± 16.16	
No	306 (88.44)	103.31 ± 19.35	
*Uterinefibroid*			0.362
Yes	24 (6.94)	106.42 ± 17.68	
No	322 (93.06)	102.61 ± 19.12	
*Menoxenia*			0.982
Yes	45 (13.01)	102.67 ± 19.06	
No	301 (86.99)	102.91 ± 19.05	
*Gain weight*			0.319
Yes	112 (32.37)	101.38 ± 19.08	
No	234 (67.63)	103.60 ± 19.00	
*Types of discomfort symptoms*			0.006**
1 species	109 (31.50)	107.34 ± 17.50	
2 species	87 (25.14)	103.64 ± 18.43	
3 species	62 (17.92)	102.34 ± 19.95	
4 species	45 (13.01)	101.00 ± 18.23	
5 species	20 (5.78)	88.60 ± 24.71	
≥ 6 species	23 (6.65)	96.39 ± 14.60	
*The years of diagnosis*			0.017*
≤ 5	298 (86.13)	101.87 ± 19.60	
> 5	48 (13.97)	109.15 ± 13.52	
*The duration of medication*			0.019*
< 3	175 (50.58)	100.57 ± 19.80	
3–6	145 (41.91)	104.17 ± 18.35	
> 6	26 (7.51)	111.27 ± 14.42	
*Psychosocial adaptation*			0.000**
≤ 38	137 (39.60)	87.64 ± 16.73	
> 38	209 (60.40)	112.87 ± 12.81	
*Medication adherence*			0.004**
Low adherence	131 (37.86)	99.15 ± 19.24	
Moderate adherence	135 (39.02)	103.33 ± 19.70	
High adherence	80 (23.12)	108.23 ± 16.17	

^*^P < 0.05, **P < 0.01

The total score of quality of life in patients with breast cancer undergoing adjuvant endocrine therapy was 102.88 ± 19.03, while the scores in the five areas of physical wellbeing, social/ family well-being, emotional well-being, functional well-being and additional concerns were 22.61 ± 3.72, 19.04 ± 5.63, 18.48 ± 4.26, 19.35 ± 5.82, 23.40 ± 5.04, respectively (Table 2).

**Table 2:** Patients’ quality of life

Item	N	Minimum	Maximum	Mean	SD
Physical well-being	346	10	28	22.61	3.72
Social/family well-being	346	1	28	19.04	5.63
Emotional well-being	346	3	24	18.48	4.26
Functional well-being	346	4	28	19.35	5.82
Additional concerns	346	2	34	23.40	5.04
Total QOL score	346	28	140	102.88	19.33

The results of univariate analysis of QOL showed that there were significant differences in QOL among patients with different types of exercise, medical payment methods, discomfort symptoms (headache, hypomnesis, arthralgia, perturbation), combination of discomfort symptoms, medication adherence and psychosocial adaptation (p < 0.05). Patients with regular exercise, urban basic medical insurance, no uncomfortable symptoms, fewer combinations of discomfort symptoms, good adherence and PSA also had a higher QOL (Table 1).

The variables with statistical significance in the univariate analysis were taken as independent variables and the QOL as dependent variables. The stepwise regression method was adopted. The assignment methods of independent variables: for binary variables, "No" is taken as the reference; for medical payment methods, " Self-pay" is taken as the reference; for ordinal categorical variables, values are assigned in order of their rank; for continuous variables, original data are entered. The result showed that PSA and medication adherence were the influential factors of QOL (p < 0.05), and PSA had a greater effect. The equation model R^2^ = 0.593 (p < 0.001), it can be considered that the factors in this model explain 59.3% of the total variation in the QOL of BCW with AET. The multivariate stepwise regression results of the influencing factors of QOL are shown in Table 3.

**Table 3:** Multivariate stepwise regression results of influencing factors of QOL

	Partial regression coefficient B	Standard error	Standardized regression coefficient Beta	t	p
Constant term	40.522	3.430		11.813	0.000
Psychosocial adaptation	1.382	0.064	0.754	21.638	0.000
Medication adherence	0.910	0.406	0.078	2.240	0.026

R^2^ = 0.593. F = 250.093, p < 0.001

**Fig. 1:**
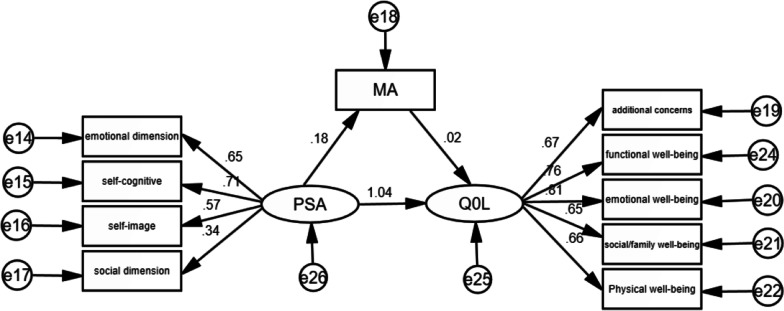
Structural model of standardized medication adherence as mediator

To explore the mediating relationship between medication adherence and PSA and QOL, we tried to introduce SPSS process plug-in with PSA as the independent variable, QOL of breast cancer women as the dependent variable and medication adherence as the intermediary. The results showed that PSA had a statistically significant impact on quality of life (β = 1.41, p < 0.01). The effect of PSA on medication adherence was statistically significant (β = 0.02, p < 0.01). PSA and medication adherence had statistically significant effects on QOL (β = 1.38, p < 0.01; β = 0.91, p < 0.05). Medication adherence partly mediated PSA and QOL. The structural model of standardized medication adherence as mediation was drawn in Amos software, as shown in Fig. 1.

Bootstrap test was used to verify the mediating effect of medication adherence. The results showed that the indirect effect of PSA on the QOL through medication adherence was 0.022, the intermediate effect accounted for 1.57%, that is, the contribution of the mediating effect to the total effect reached 1.57%, and the Bootstrap 95% CI (0.0025,0.0584), the interval did not include 0, Therefore, the effect of mediating effect is statistically significant. The results of Bootstrap mediation are shown in Table 4.

**Table 4:** Bootstrap results of mediation effects

Effect of type	Effect size	Boot	Bootstrap 95%CI		Relative effect ratio
		SE	The lower limit	The superior limit	
The total effect	1.405	0.064	1.280	1.530	–
Direct effect	1.383	0.064	1.257	1.508	98.43%
Indirect effect	0.022	0.014	0.003	0.058	1.57%

## Discussion

The BCW in this survey is mainly concentrated in the middle-aged population (the average age is 50.46 ± 9.23), which is consistent with the existing research conclusion that breast cancer in China is most common in the 45–50 years old and 60–65 years old [16, 17]. Medical payment methods are mainly urban basic medical insurance and cooperative medical care, accounting for 77.46% and 14.45% respectively. This may be due to the fact that most of the subjects in this study are urban residents, and the coverage area of the national medical insurance benefits is relatively wide. This result is basically consistent with other studies [7, 18–21]. About 47.40% of breast cancer women undergoing endocrine therapy developed hot flashes, about 46.82% developed hypomnesis, about 39.88% developed joint pain, and 35.84% developed joint stiffness. With the increase of the combination types of discomfort symptoms, the corresponding number of patients decreased. Only one uncomfortable symptom accounted for about 31.50%, and the combination of 6 or more uncomfortable symptoms accounted for about 6.65%. The occurrence of these symptoms may be related to the adverse reactions of endocrine drugs taken by patients themselves, or the disease itself accompanied with symptoms [22–24]. About 86.13% of the subjects were diagnosed for less than 5 years, which may be related to the fact that the subjects in this study are mainly young and middle-aged, or it may be the reason that women do not pay attention to breast cancer physical examination, or the positive rate of physical examination is low. Medication time < 3 years were accounted for about 50.58%, the medication time 3–6 years accounted for about 41.91%, and the medication time > 6 years accounted for about 7.51%. This may be related to the increasing rate of medication discontinuation over time, or to the younger age of the study subjects and the common practice of clinical endocrine therapy for 5 years. Studies have shown that women taking a single drug for breast cancer are more likely to stop taking their medication over a period of two to three years [25, 26].

In this study, all dimensions of psychological and social functions of breast cancer women treated with endocrine therapy and their overall status were at a moderately good level. The reasons may be related to the good family support and social relations, the improvement of education level and the change of self-value perception of breast cancer women [27, 28]. from scale entry mean score compared with the overall mean alone, the items "breast problems make me feel ugly" and "other people sometimes make unpleasant comments about my disease" had average scores lower than the mean of the total items (2.56), indicating that patients have a lower sense of self-identity and are more influenced by the attitudes of others toward them; the items "Breast problems affect my sex life as a couple," "I try to keep trying even when I'm failing," and "I try to see things in a positive light," averaged higher than the overall mean, indicating that breast cancer women were better at having sex and coping with difficulties in a positive manner.

Poor medication adherence accounted for 37.89%, and medium medication adherence or above (medication adherence rate) accounted for 62.14%, indicating that the vast majority of patients had good medication adherence, which was consistent with the results of a review report [10]. In this study, the proportion of high adherence was only 23.12%, lower than the result of Kesmodel SB et al. [29]. In their study, the proportion of high adherence was 50%, which may be related to the long time span of medication in this study. Some studies have pointed out that the longer the medication time, the worse the medication adherence may be [26]. "Have you ever forgotten to take your endocrine medication?", "Have you ever been without endocrine medication?", "Have you ever gone outside without taking endocrine medication?" The average scores of the three items was lower than the overall mean of 0.79, indicating that patients forgot to take medicine, did not take endocrine drugs within 2 weeks, and medication leakage had negative effects on medication adherence. In addition, other studies have pointed out that elderly women have high medication adherence, while the majority of the population in this study was young and middle-aged women, which may also be one of the reasons for the small proportion of high adherence in this study [30]. Although in this study, good medication adherence accounted for 62.14%, which was above the average level, but high medication adherence accounted for a relatively low proportion. Therefore, measures should be taken to further improve medication adherence.

In this study, the average score of quality of life was 102.88. Although the overall quality of life of the subjects was good, about 43.06% of the subjects still scored lower than the mean. The total mean score of each item in the scale was 2.86.14 items: "Side effects of treatment make me feel bad”, "Hair loss makes me worry", "my friends and I very close to", "I am happy with my family and talk about my illness", "I am satisfied with my sex lives", "I feel sexually attractive", "I have a good sleep", "I worry about the effect of nervousness on my illness", "I was worried that someone else in my family might one day get the same disease as me", "I am able to work ( including work from home), "my work (including work from home)makes me feel fulfilled.", "I am proud of how I treat my illness.", "I care about how I dress."," I can still feel like a woman" scores are below average. It shows that the side effects of treatment, alienation of social and family relations, poor experience of sexual relationship, tension and worry, low sense of job achievement, the disease of shame, and body image disorder are not good for the improvement of patients' quality of life, and the poor sexual life is the most prominent one. In addition, Although the PSA score showed that most subjects showed a positive attitude towards sexual life after endocrine therapy, the quality of life in real life was seriously affected by the negative impact of sexual life, that is, the quality of sexual life decreased, which was also proved in the studies of scholars such as Andrzejcza [31]. Thus it can be seen that endocrine therapy of breast cancer women psychological change in sex life trajectory, on the one hand, active internal adjustment to adapt to sexual life changes, on the one hand, face the reality of low sexual experience, suggest we nursing intervention or psychological education in the future, not just for women with only one side, at the same time should also make their spouses accepted and recognized the physiological changes of the other party, and then accept each other.

In single factor analysis, exercise had a positive impact on quality of life, that is to say, the quality of life of regular exercisers is higher than that of irregular exercisers, while the quality of life of irregular exercisers is higher than that of non-exercisers. It may be because regular exercise can exercise the patients' physiological functions, promote the body's recovery, and thus improve the quality of life of patients as a whole [32–35]. The study showed that, different payment methods of medical expenses have an impact on the difference in QOL, and different reimbursement rates of medical expenses will bring different economic pressures to patients, which will affect patients' drug noncompliance, and then lead to the difference in QOL [36]. Symptoms can significantly interfere with the patients' quality of life, this research shows that the greater the number of different types of symptoms that patients themselves present, the worse their quality of life, and symptoms of harmful to the quality of life is to have a headache,, joint pain, hypomnesis and other symptoms are the most prominent,and Chan et al., study result is the same [37]. It is suggested that in the medication and nursing of patients, attention should be paid to the use of drugs with less adverse reactions or to develop personalized nursing intervention measures for the relevant symptoms of discomfort, in order to alleviate or avoid the discomfort of patients; At the same time, medical staff should be actively trained on specific symptoms related to endocrine therapy, enhance their attention, strengthen the assessment and management of related symptoms, and actively take preventive measures to reduce uncomfortable symptoms [20, 38, 39]. The diagnosis time and taking the longer the duration, quality of life scores worse, studies have pointed out that five years endocrine therapy of breast cancer treatment effect is obvious, the recurrence rate is low, but side effects are increased [40]. The reason why the quality of life of patients decreased with the increase of medication duration may be due to the gradual prominence of drug side effects and the decrease of medication compliance with the extension of medication duration [41].

Multi-factor analysis showed that psychosocial adjustment and medication compliance were the influencing factors of quality of life. As the secondary sexual organ of women, breast plays an important role in women's physical beauty, and the change of breast will lead to stigma and change of body image [42, 43], and positive PSA can improve QOL. Studies have found that negative psychological will severely hampered the patient's quality of life [44], suggesting that we make in the future nursing measures should be conducive to improve the patient's mental state, optimize personal expectations, help them to establish correct disease cognition and self-awareness, reduce the psychological burden and emotions, to encourage patients to establish a good attitude, positive when it is necessary to give psychological health education, help the patient accept or adapt to changes in the body [45]. Medication compliance is also an important factor affecting quality of life, and the higher the medication compliance, the better the quality of life, as demonstrated in a community-based cohort study [46]. Therefore, targeted interventions should be made for the influencing factors of medication compliance, such as use of combined drug delivery, conduct discharge education, provide medication guidance and adverse reactions, emphasis on the importance of medication, establishment of a good follow-up system, and development of intelligent medication reminder service to improve medication compliance [21, 47].

In this study, SPSS Process was used to examine the mediating effect of medication compliance on psychosocial adjustment and quality of life of breast cancer women undergoing endocrine therapy, and Bootstrap test was used to verify the mediating effect of medication compliance. The results supported the mediating effect of medication compliance. In addition, the total score of PSA has a significant direct effect on the quality of life, occupying a major position, while the indirect effect of PSA on the quality of life through medication compliance is 1.57%, this indicates that patients with good PSA can improve their quality of life to a certain extent by improving their medication compliance. Although the role of the mediating variable of medication compliance in the indirect impact of PSA on quality of life is weak, it is statistically significant, so the existence of this role cannot be denied [48]. This also suggests that we should pay more attention to the improvement of patients' PSA in the future clinical nursing work, which can improve the quality of life of patients more directly and quickly.

### Limitations of research

All the patients included in this study were only from two hospitals in northeast China, which is less than the number of breast cancer patients in the whole country, and the influencing factors such as regional culture and economic status were not fully considered. Secondly, this study was a cross-sectional study, unable to show the dynamic development trajectory of quality of life, psychosocial adjustment and medication compliance of breast cancer women undergoing endocrine therapy with the change of disease years, medication years and other factors. The related longitudinal development will be further carried out in the future. In addition, medication compliance in this study was measured by a self-filled questionnaire without objective indicators, such as electronic medication monitoring system, biomarkers, etc.

## Conclusions

The quality of life of patients with breast cancer endocrine therapy will be directly affected by PSA. Medication compliance has a weak mediating effect in the indirect impact of PSA on the quality of life. In the future, clinical nursing work should take targeted measures to improve the PSA level of patients, and effectively improve the compliance of patients with medication, so as to better improve the quality of life of breast cancer survivors.

## Data Availability

The datasets used and analysed during the current study available from the corresponding author on reasonable request.

## Abbreviations

AET: Adjuvant Endocrine Therapy
BCW: Breast Cancer Women
ER: Rstrogen Receptor
IARC: International Agency for Research on Cancer
PR: Progesterone Receptor
PSA: Psychosocial Adaptation
QOL: Quality of Life
WHO: World Health Organization
